# Sulfuric Acid Catalyzed Esterification of Amino Acids in Thin Film

**DOI:** 10.1021/jasms.3c00284

**Published:** 2023-10-31

**Authors:** Chiara Salvitti, Giulia de Petris, Anna Troiani, Marta Managò, Alessia Di Noi, Andreina Ricci, Federico Pepi

**Affiliations:** † “Sapienza” University of Rome, Department of Chemistry and Drug Technologies P.le Aldo Moro 5, 00185 Rome, Italy; ‡ Department of Mathematics and Physics, University of Campania L. Vanvitelli, Viale Lincoln 5, 81100, Caserta, Italy

**Keywords:** ESI mass spectrometry, microdroplets, thin film, amino acids, esterification

## Abstract

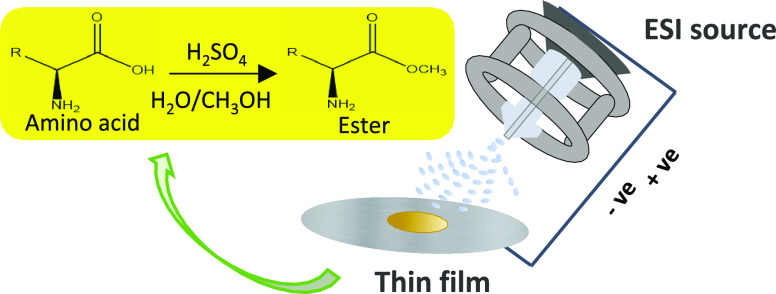

The esterification reaction of different amino acids with methanol catalyzed by H_2_SO_4_ was first studied in the small volume of thin films generated by ESI microdroplet deposition. The reaction is promoted by the pneumatic spray of the ESI source and reaches its maximum efficiency at a thin film temperature of 70 °C. Selective esterification of the COOH moiety was demonstrated. Microdroplet size and thin film volume and lifetime are critical parameters that influenced the reaction outcome. As expected, l-tyrosine and l-phenylalanine having aromatic side chain substituents were the most reactive amino acids, reaching absolute yields of around 40–50%. The amino acid esterification catalyzed by H_2_SO_4_ in a thin film occurs under synthetic conditions in which the same reaction in the bulk is not observed.

## Introduction

Amino acids are the building blocks of proteins, hormones, and neurotransmitters in living organisms. Amino acid deficiencies can be treated by dietary supplementation, but amino acid based drugs generally have a low bioavailability due to low intestinal permeability, extensive metabolism, and rapid liver clearance. Thus, their chemical derivatization is the main approach of the prodrug strategy.^1,2^ Moreover, the presence of a carboxyl function, an amino group, and eventually aromatic, amino, carboxyl, and hydroxyl additional groups in a single molecule offers a large variety of possibilities for fine-tuning the final prodrug properties. In this view, amino acid esters are widely used in food and pharmaceutical industries as amino acid precursors.^3−8^ In particular, the pharmaceutical application of amino acid esters as prodrugs demonstrates that esterification may solve problems associated with their low solubility and instability. Amino acid esters are also used in food industries to improve food flavor and extend product shelf life. Furthermore, conventional peptide synthesis and protein analytical protocols often take advantage of amino acid esterification to protect the −COOH group.^9^ Amino acid esters are synthesized by using a large variety of different catalysts starting from classical mineral acids, such as HCl and H_2_SO_4_, up to ionic liquids, zeolites, microwaves, and enzymes.^10−20^ Rare-earth doping of solid superacids has been used to prepare various ester compounds, and Nd doping was recently employed for aromatic amino acid esterification.^21^ The esterification reaction is usually conducted in the presence of the corresponding alcohol as an organic solvent, and the continuous removal of water is required to push the reaction equilibrium forward. Moreover, esterification of amino acids is much more difficult than that of ordinary carboxylic acids due to their zwitterionic structure, and the reaction yields are often low due to the concomitant amino group alkylation that requires tedious N-protection, esterification, and deprotection workup procedures.

In the last few years, several milestone reactions of organic chemistry have been studied in the microdroplets generated into the electrospray ionization (ESI) source of a mass spectrometer.^22−29^ It has been demonstrated that in the confined volume of the charged microdroplets the reaction rates are accelerated up to 10^5^ times compared with the same process in bulk. Moreover, the microdroplets landing onto a solid surface generate a thin film by which the neutral products of the reaction can be separated and quantified.^30−34^ The thin film retains the peculiar confined volume of the microdroplets needed for reaction acceleration, but it is characterized by a longer lifetime that allows one to extend the reaction time to any desired value. Low molecule solvation, formation of highly reactive transient ionic species, extreme pH value, and a high interfacial electric field are some of the parameters invoked to explain the strong acceleration factors observed in confined volumes with respect to the same processes in bulk.^35−38^ From this point of view, the carboxylic acid esterification reaction, similarly to other dehydration reactions,^39−44^ may benefit from the peculiar fast solvent evaporation operative in the microdroplet/thin film system. Nevertheless, among the large number of organic reactions accelerated in the microdroplet environment, the only esterification example was recently reported by Pradeep et al. that demonstrates the esterification of fatty acids with sugars at the interface of two immiscible liquid microdroplet beams.^45^

In this study, the esterification of different amino acids in a thin film was investigated by using different mixtures containing natural amino acids and H_2_SO_4_ as the acid catalyst. The reaction products were structurally characterized by collision-induced dissociation (CID) mass spectrometry. The efficiency of the observed reaction was evaluated in terms of conversion ratio (CR), and absolute yields were also estimated by using ionization efficiency calibration curves. The dependence of the conversion ratio on fundamental ESI parameters such as cone voltage polarity and desolvation gas temperature was also evaluated.

## Experimental Section

### Reagents

l-Alanine, l-phenylalanine, l-tyrosine, l-lysine, and l-glutamic acid and their methyl esters, HCl, H_2_SO_4_, HNO_3_, solvents, and all other chemicals were purchased from Sigma-Aldrich Ltd. and used without further purification.

### Mass Spectrometric Experiments

Microdroplet deposition experiments were performed by using the Z-spray electrospray ionization (ESI) source of a quadrupole-time-of-flight (Q-TOF, Ultima) mass spectrometer (Micromass, Manchester, UK) suitably adapted to thin film reaction studies.^46^ Briefly, in the ESI Z-spray source, the ions dried by the N_2_ desolvation gas and deflected by the sampling cone voltage enter into the in-vacuum analyzers of the mass spectrometer to be mass analyzed. At the same time, the charged microdroplets that flew in-line hit a stainless steel surface whose distance from the exit of the ESI capillary can be varied from 2 to 3.2 cm (Figure S1).

Millimolar starting solutions were prepared by dissolving l-amino acids in 1:1 H_2_O/CH_3_OH solvent mixtures and by adding an inorganic acid catalyst (H_2_SO_4_, HCl, or HNO_3_). The use of buffers was avoided to prevent any interference with the mass spectrometric analysis. The solutions thus obtained were infused into the ESI source of the instrument, and the ionic composition of the microdroplets was online-analyzed before performing the deposition experiments. Nitrogen was used as a desolvation gas at a flow rate of 200 L h^–1^, whereas the source and desolvation temperatures were set at 80 and 150 °C, respectively. Typical source potentials are as follows: capillary 4 kV, cone 60 V, RF lens-1 70 V, and syringe pump flow of 20 μL min^–1^. The displayed mass spectra were obtained by averaging 100 scans in the 50–500 *m*/*z* range. After acquiring the zero-time mass spectrum, 1 mL of each 1:1 H_2_O/CH_3_OH 1 × 10^–3^ M acidic solution of the five different amino acids was injected into the ESI source, and the charged microdroplets were collected onto the stainless-steel plate held 3.0 cm away from the capillary tip. Taking the amount of the amino acid constant, two different solutions with 3:1 and 1:3 H_2_SO_4_/amino acid ratios having pH values of 2.0 and 2.7, respectively, were used.

Microdroplet deposition leads to the formation of a thin film from which a solid precipitate separates at the end of the deposition time after spontaneous solvent evaporation. The solid precipitate was rinsed with 1 mL of the same sprayed solvent mixture, and the resulting solution was mass-analyzed under the same ESI experimental conditions used to acquire the zero-time mass spectrum. To test the reproducibility of the system, three independent experiments were performed for each amino acid on different days.

Rough estimates of the thin film synthetic yields were obtained by measuring the conversion ratio (CR) following the general formula [P1]/[R], where [P1] and [R] are the ionic intensities of the thin film product and reagent, respectively. Changes in the conversion ratios were in turn evaluated as a function of the source parameters by varying the capillary voltage polarity (+4.5 kV, −3.5 kV, 0), and desolvation gas temperature (80, 150, and 300 °C). Absolute yields were derived by measuring the ionization correction factors. To this end, different solutions containing variable amounts of the standard amino acid and its methyl ester were prepared and mass analyzed under the same ESI experimental conditions used to analyze the thin film reaction products. The ratio of the measured ionic intensities of the amino acid and ester protonated species were hence plotted versus the theoretical ratio of the prepared standard solutions and ionization correction factors derived by the equation of the linear regression line obtained (see below).

To measure the apparent acceleration factors of the thin film reactions, the intensity ratios between products and reagents from the droplet deposition process were compared to the corresponding values measured for the reactions performed in bulk. In a vessel, each 10^–3^ M amino acid H_2_O/CH_3_OH (v/v, 1:1) solution containing H_2_SO_4_ at pH = 2 was stirred with a magnetic bar and heated at 100 °C under reflux. After 1 h, the reaction was quenched and the mixture cooled to room temperature. An aliquot was then sampled and subjected to mass spectrometric analysis using the same ESI experimental parameters previously reported. The reactions were also tested in a static thick film obtained by dropping 1 mL of the same solutions onto a stainless-steel surface and allowing evaporation of the solvents (water/methanol) at 70 °C in a thermostatic oven. Also in this case, the precipitate separated from the thick film was rinsed with the same solvent mixture and analyzed using the same ESI experimental conditions previously described.

The reaction products and intermediates were characterized by collision-induced dissociation (CID) experiments. CID mass spectra were acquired by introducing Ar as the target gas into the quadrupole cell at pressures of about 0.1–0.5 mTorr. Data acquisition and processing were carried out by using MassLynx version 4.0 software supplied with the instrument.

## Results and Discussion

**Figure 1:**
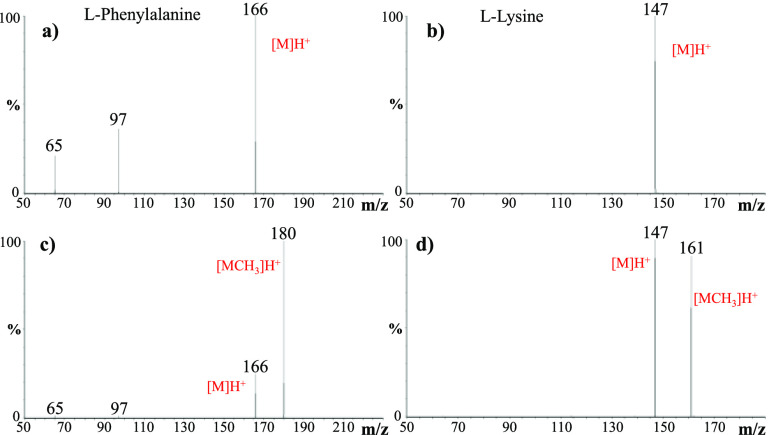
Positive ESI mass spectra of the starting solutions containing (a) l-phenylalanine and (b) l-lysine dissolved in a 1:1 H_2_O, H_2_SO_4_ (pH = 2)/CH_3_OH mixture at a concentration of 1 × 10^–3^ M. Positive ESI mass spectra of the thin film products formed from (c) l-phenylalanine and (d) l-lysine systems. Protonated amino acids and the corresponding methyl esters are indicated as [M]H^+^ and [MCH_3_]H^+^, respectively. Ions at *m*/*z* 65 and 97 refer to [(CH_3_OH)_2_]H^+^ and [(CH_3_OH)_3_]H^+^ methanol adducts, respectively.

Thin film amino acid esterification with methanol was studied by spraying onto a target stainless-steel surface the solutions of five different amino acids (l-alanine, l-phenylalanine, l-tyrosine, l-lysine, and l-glutamic acid) in the presence of different amounts of H_2_SO_4_ as a mineral acid catalyst. The selected reactants were chosen as being representative of differently side chain substituted amino acids, having additional alkyl, aromatic, amino, and COOH functional groups. The mass spectra of the l-phenylalanine and l-lysine solutions in the presence of H_2_SO_4_ catalyst at pH = 2, chosen as model systems, are displayed in Figure 1, whereas all the mass spectra of the other amino acid solutions tested are reported in Figures S2–S4 in the Supporting Information. As is evident in Figure 1a,b, the ESI positive mass spectra of the starting H_2_SO_4_ pH = 2 solutions are dominated by the amino acid MH^+^ ion at *m*/*z* 166 and 147, respectively. Intense ionic signals corresponding to methanol protonated clusters are also present in the spectrum of l-phenylalanine, whereas the presence of an additional ε-NH_2_ basic group in l-lysine strongly favors the formation of its protonated species. No ionic species attributable to the amino acid esterification were observed, thus demonstrating that no reaction occurs in the microdroplets before their deposition onto the solid surface.

The deposition of the ESI microdroplets onto the stainless-steel surface generates a stable thin film from which a yellow precipitate separates during the reaction time (typically 50 min). Besides the amino acid MH^+^ ions, the ESI mass spectra of the precipitates rinsed with the H_2_O/CH_3_OH solvent mixture show additional ions at *m*/*z* 180 and 161 formally corresponding to the addition of a methyl group to l-phenylalanine and l-lysine, respectively (Figure 1c,d). Similar results were obtained for all of the other investigated amino acid systems (Figures S2–S4). In the case of l-glutamic acid, the concomitant formation of mono- and dimethylated products was observed (Figure S4) likely coming from the esterification of its two COOH functional groups. Analogous thin film reaction experiments were also conducted by depositing the microdroplets produced in negative ESI ion mode or without the potential applied to the ESI needle. For direct comparison purposes, the rinsed precipitate was always analyzed in positive ESI ion mode that showed good signals for both the free and esterified amino acids.

To probe whether the observed products correspond to the amino acid −COOH methyl ester, the CID mass spectra of the thin film products were compared with those of the commercially available standards (Figures S5–S8). Apart from the l-alanine methyl ester that does not fragment upon collision with Ar (Figure S8) and l-lysine methyl ester that fragments after losing a NH_3_ molecule (Figure S6), the loss of a CH_3_COOH moiety seems to be the peculiar fragmentation, indicating the esterification of the COOH group. The CID mass spectra of the products derived from the thin film reactions showed these fragmentations, and all were superimposable with those of the corresponding standard amino acid methyl esters.

**Figure 2:**
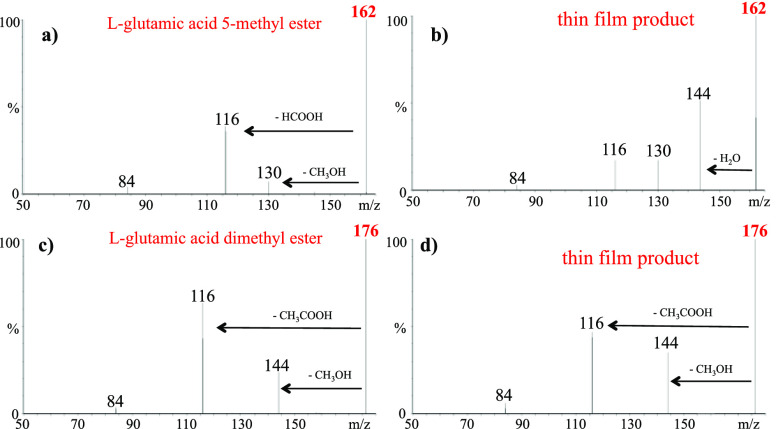
(a) CID mass spectrum of the standard compound l-glutamic acid 5-methyl ester. (b) CID mass spectrum of the l-glutamic monomethyl ester product formed by the reaction in the thin film. (c) CID mass spectrum of the standard compound l-glutamic dimethyl ester. (d) CID mass spectrum of the l-glutamic dimethyl ester product formed by the reaction in the thin film. The *m*/*z* ratios of the protonated parent species are indicated in red. Major fragment ions are highlighted with black arrows.

In the case of l-glutamic acid, the CID mass spectrum of the standard l-glutamic acid 5-methyl ester (Figure 2a), corresponding to the amino acid esterified on the side chain COOH group, is characterized by the loss of methanol and HCOOH, whereas the monoester obtained from the thin film reaction, besides these fragmentations, also shows the loss of a water molecule (Figure 2b). The losses of water and formic acid are the characteristic fragmentations of l-glutamic acid (Figure S9), and hence it is reasonable to assume that l-glutamic acid thin film monoesterification occurs on both of the amino acid COOH moieties. The CID of standard l-glutamic acid dimethyl ester is superimposable with that of the ionic species derived by the thin film reaction (Figure 2c,d, respectively), showing the common loss of CH_3_COOH and the minor loss of methanol peculiar of the COOH side chain group esterification.

A first estimation of the efficiency of the esterification reaction observed was obtained by measuring the conversion ratios (CRs) of all of the investigated amino acid thin film systems that are summarized in Table 1. Very low conversion ratios were measured for the less acidic solutions at pH = 2.7, whereas conversion ratios of around 80–90% were measured for most of the amino acids at pH = 2, except for l-lysine, whose CR is around 50% in positive and negative ion modes and increases to 77.2% without the applied cone voltage. Each CR value reported represents the mean of three independent measurements. It is interesting to underline that the ESI capillary voltage does not interfere with the reaction yields even in the positive and negative polarities. Moreover, the experiments performed without the applied voltage showed always a slightly higher reaction efficiency. This effect could be justified by the formation of larger microdroplets whose deposition leads to the formation of a larger thin film volume clearly visible even to the naked eye, within which the molecules may continue to react until the solvent is completely dried.

**Table 1:**
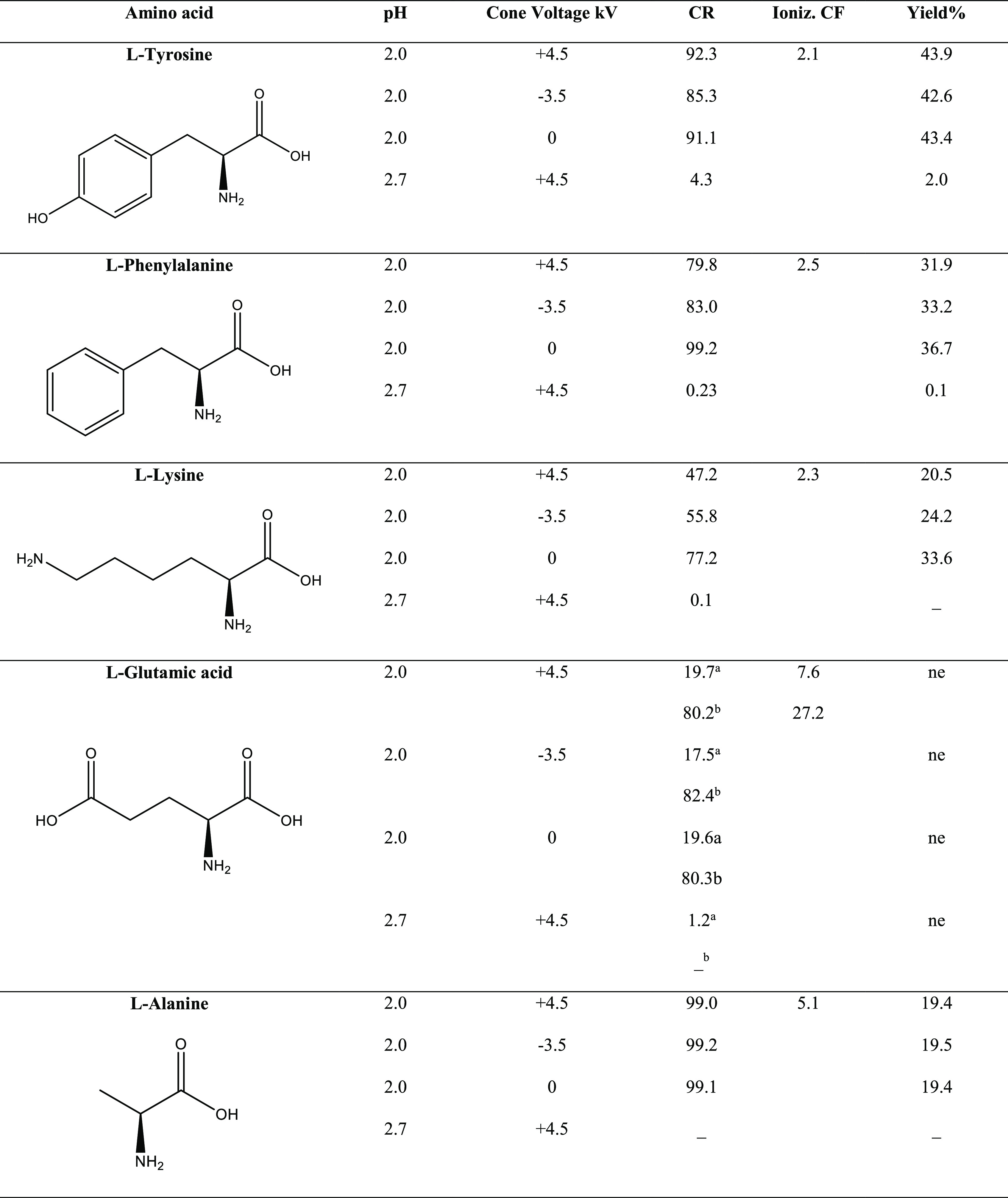
Thin Film Conversion Ratios (CRs), Ionization Correction Factors (CFs), and Reaction Yields of the H_2_SO_4_-Catalyzed Amino Acid Esterification at Different pH Values and ESI Cone Voltage Potentials^c^

a l-Glutamic acid monoester.

b l-Glutamic acid diester.

c ne = value not estimated, see text.

It should be emphasized that the conversion ratios only allow a rough estimation of the reaction yields, since they are strongly influenced by the ionization efficiency of reactants and products. From this point of view, it is legitimate to suppose that the real yields of the reaction should be better reproduced by the conversion ratio measured for amino acids having an additional basic group, as in the case of l-lysine, l-phenylalanine, and l-tyrosine, whose basicity should be scarcely influenced by the presence of the methyl substituent on the carboxyl function. To probe this assumption, the ionization efficiency of each methyl ester with respect to the free amino acid was derived by the ratio of ester (E) and amino acid (A) protonated ion intensities measured in the ESI mass spectra of mixtures containing variable quantities of the two molecules (named R_E/A_ experimental).

**Figure 3:**
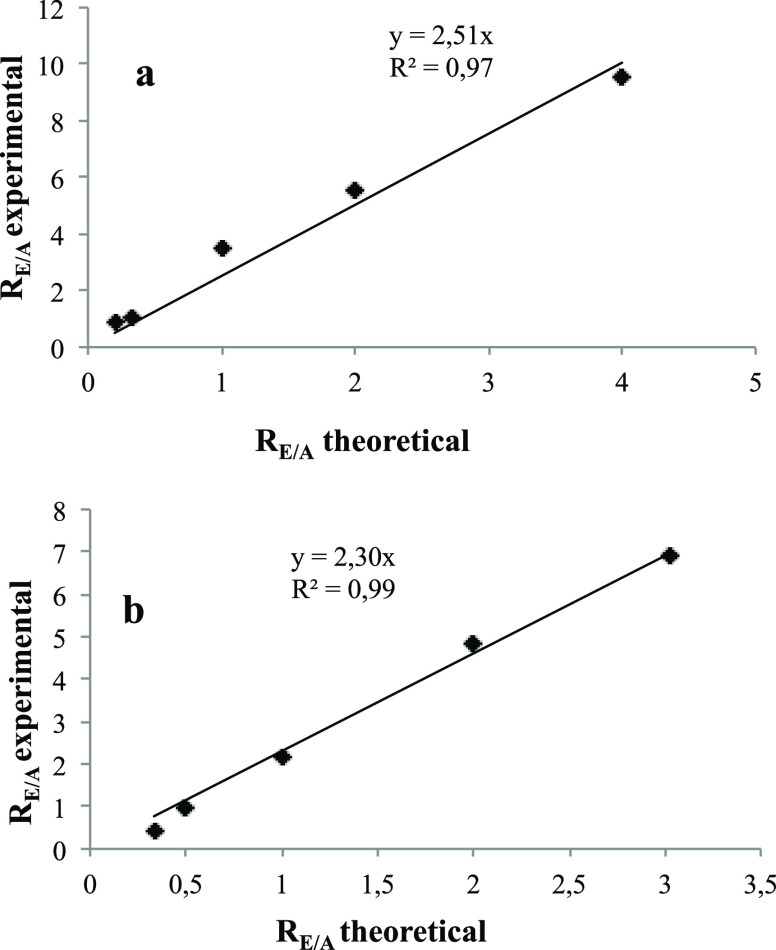
(a) l-Phenylalanine and (b) l-lysine calibration curves built by plotting the ratios of known amounts of standard amino acid (A) and the corresponding methyl ester (E) named R_E/A_ theoretical and the protonated ester and amino acid ion intensities denoted as R_E/A_ experimental. The linear regression analysis is reported in each graphic.

Calibration curves were hence obtained by plotting these values versus the theoretical ratio of the mixed standard molecules (named the R_E/A_ theoretical). l-Phenylalanine and l-lysine calibration curves are shown in Figure 3, whereas the plots obtained for the other amino acids are reported in Figures S10–S13 in the the Supporting Information. The plots are all characterized by linear regression lines with *R*^2^ exceeding 96%, and the ionization correction factors (CFs) derived were used to estimate the absolute yields reported in Table 1.

l-Lysine, l-phenylalanine, and l-tyrosine show ionization efficiency correction factors of around 2 leading to absolute reaction yields of around 40% for l-tyrosine and l-phenylalanine, whereas yields around 25–30% can be estimated for l-lysine. As supposed, larger correction factors were measured for amino acids without additional basic groups, as in the case of l-alanine, or characterized by additional acidic substituents, as in the case of l-glutamic acid. Moreover, the very low ionization efficiency of free l-glutamic acid with respect to its esters required using ester/amino acid ratios in favor of the amino acid in the ionization efficiency calibration curves (Figure S13). This condition is opposite with respect to the ionic signal measured in the thin film experiments, where the free amino acid has a relative intensity never exceeding 0.1% with respect to the sum of its esters (Figure S4). For this reason, the CR relative to l-glutamic amino acid was not corrected, since the values eventually obtained contain a large error and are not representative of its actual esterification yields. Moreover, absolute yields of l-glutamic acid esterification in line with the values obtained for the other amino acids (around 50%) can be certainly assumed by taking into account that going from a 1:1 to a 2:1 ratio in favor of the ester the peak relative to the protonated amino acid is no longer detectable.

### Effects of the Thin Film Temperature

ESI source parameters, such as spray temperature, may strongly affect the volume and the lifetime of the thin film obtained by the microdroplet deposition.^47^ In the instrumental setup used, the temperature of the thin film resulting from microdroplet deposition depends on the nitrogen desolvation gas temperature of the source block. This parameter was varied from 80 to 300 °C, and the corresponding actual spray temperatures were measured by a thermocouple positioned onto the stainless-steel target at the point where the microdroplets are deposited, generating the thin film. Commonly, the actual thin film temperature measured was around 50% of the set desolvation gas temperature, i.e. the desolvation gas temperature of 150 °C used for the experiments described above corresponds to an actual thin film temperature of 70 °C. The measured conversion ratio at nominal desolvation gas temperatures of 80, 150, and 300 °C for the model l-phenylalanine and l-lysine amino acid solutions sprayed in an ESI positive condition are reported in Table 2. It is interesting to note that the increase of the desolvation gas temperature from 150 to 300 °C leads to a drastic reduction in the esterification yields, whereas the conversion ratios measured are only slightly lowered by reducing the temperature to 80 °C. It is reasonable to assume that at higher temperatures the fast desolvation of the microdroplets gives rise to the deposition of a smaller thin film volume which dries quickly in solid-state reactants and products, thus preventing the progress of the reaction. The optimal thin film reaction temperature is around 70 °C.

**Table 2:** Thin Film Conversion Ratios (CRs) Measured at Different Temperatures

amino acid	desolvation gas temp (°C)	thin film temp (°C)	CR
l-phenylalanine	80	40	59.7
	150	70	79.8
	300	160	8.2
			
l-lysine	80	40	26.2
	150	70	47.2
	300	160	7.1

### Effects of the Acid Catalyst

The amino acid esterification was also tested by using solutions containing HCl and HNO_3_ as acid catalysts under the same conditions used for H_2_SO_4_ (pH = 2). Interestingly, no reaction products were observed, thus indicating that not only the pH value but also the nature of the mineral acid used as a catalyst influences the esterification reaction. In solution, the ability of sulfuric acid and alkyl sulfonic acid to promote the esterification of the carboxyl group with alcohol is well-known. In fact, it has been reported that not only is the reaction mechanism acid-catalyzed but also the esterification is promoted by the alkylation of the carboxylate with sulfate or sulfonate esters that are in situ generated by the reaction of H_2_SO_4_ with the alcohol used.^48^ Nevertheless, any attempt to find possible methylsulfonate ions as reaction intermediates failed even after analyzing the reaction mixtures in negative ESI ion mode.

### Amino Acid Esterification in Thin Film versus Droplet Casting and Bulk

To establish the acceleration factors of the amino acid esterification observed in the thin film, the same reaction was performed in bulk and in a static thick film using experimental conditions comparable to those employed for the thin film experiments.

Bulk reactions were performed by heating at 100 °C under reflux for 1 h of each pH = 2 amino acid solution, whereas for the static thick film reactions, a small volume of the same solutions was dropped onto a stainless-steel surface and let dry in a thermostatic oven at 70 °C. The mass spectra of the solutions taken at time zero and after bulk and thick film reactions are completely superimposable, showing only the amino acid MH^+^ ions. No esterification reaction occurs in the bulk and in the thick film systems. The H_2_SO_4_-catalyzed amino acid esterification reaction at pH = 2 is peculiar of the thin film formed by ESI microdroplet deposition.

## Conclusions

The fast evaporation of the H_2_O/CH_3_OH solvent mixture from the thin film formed by ESI microdroplet deposition accelerates the esterification reaction of amino acids catalyzed by H_2_SO_4_. The reaction is promoted by the pneumatic spray of the ESI source, but it is not sensitive to the voltage applied to the ESI capillary and reaches its maximum efficiency at a thin film temperature of 70 °C. Microdroplet size and thin film volume and lifetime are critical parameters that influence the reaction outcome.

The use of sulfuric acid is crucial to observe the reaction, since no product was obtained by using other strong mineral acids such as HCl and HNO_3_. The esterification occurs at temperature and pH values by which the same reaction in bulk is not observed and selective esterification of the COOH moiety was demonstrated. According to the easier esterification of aromatic-substituted with respect to alkyl-substituted carboxylic acids, l-tyrosine and l-phenylalanine, having a common aromatic side chain substituent, show higher yields among the investigated amino acids. The absolute yields of the reaction were evaluated by calibrating the ionization efficiency of each amino acid with respect to its methyl ester, demonstrating that the conversion ratio usually used to estimate the yield of microdroplet reactions can be strongly affected by reagent and product ionization efficiency. Finally, the thin film synthetic procedure could be a model for other high-efficiency esterification methods proposed in solution that may occur under milder conditions if performed in confined evaporating volumes.

## Supporting Information Available

ESI Z-spray source adapted to microdroplet deposition experiments, ESI mass spectra of the amino acids in pH = 2 starting solutions compared with the ESI mass spectra of the rinsed precipitates formed after the thin film reaction, CID mass spectra of standard amino acid esters compared with the CID mass spectra of the amino acid/alcohol reaction products, and calibration curves constructed to measure the ester/amino acid ionization correction factors (PDF)
