# Supplementary material for: Sulfuric Acid Catalyzed Esterification of Amino Acids in Thin Film
Source: J Am Soc Mass Spectrom. 2023 Oct 31;34(12):2748–54. doi: 10.1021/jasms.3c00284 (PMC10704590; doi:10.1021/jasms.3c00284)
Supplement: Supplementary file 1 — js3c00284_si_001.pdf [file js3c00284_si_001.pdf]

Supporting information

Sulfuric Acid Catalyzed Esterification of Amino Acids in Thin Film

Chiara Salvitti*[a], Giulia de Petris[a], Anna Troiani[a], Marta Managò[a], Alessia Di Noi[a], Andreina Ricci[b] and Federico Pepi[a]*

[a] *"Sapienza" University of Rome, Department of Chemistry and Drug Technologies*

P.le Aldo Moro 5, 00185 Rome, Italy

[b] *Department of Mathematics and Physics, University of Campania L. Vanvitelli, Viale Lincoln 5, 81100, Caserta, Italy.*

Corresponding Authors

Prof. Federico Pepi
federico.pepi@uniroma1.it

Dr. Chiara Salvitti
chiara.salvitti@uniroma1.it

ESI SOURCE EXPERIMENTAL SET-UP

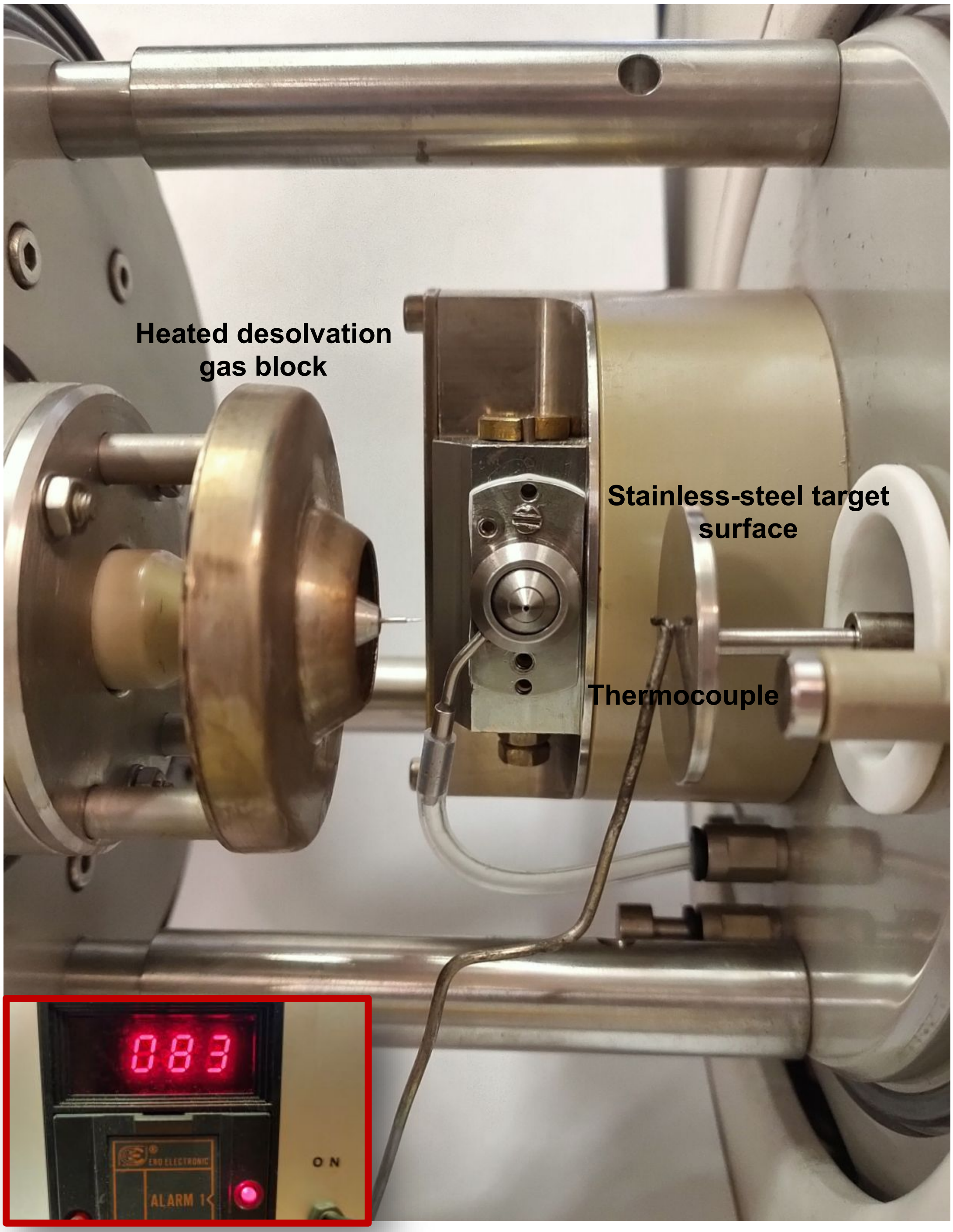

Figure S1: ESI Z-spray source adapted to the microdroplets deposition experiments.

ESI MASS SPECTRA OF REACTION MIXTURES

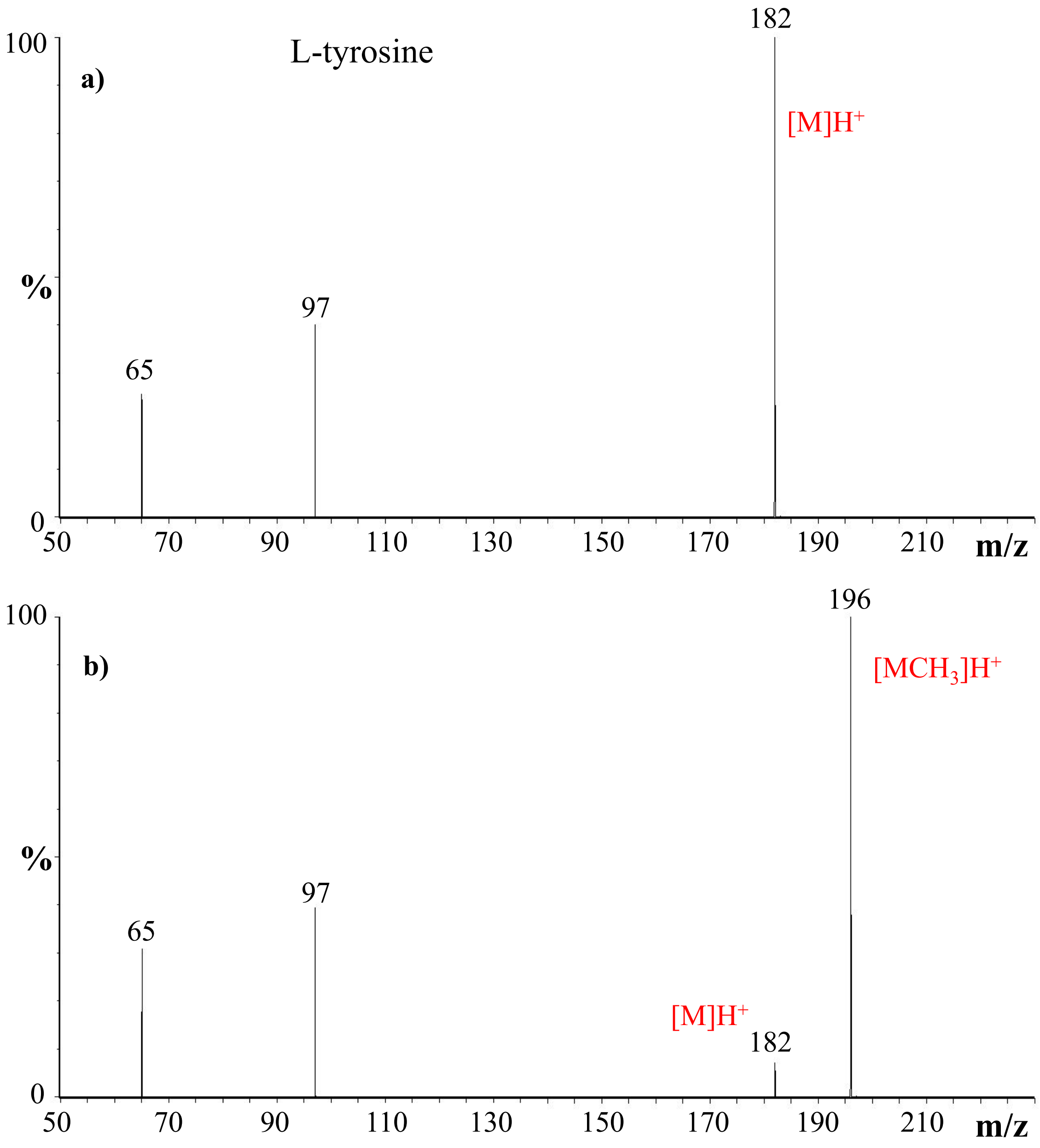

Figure S2: (a) Positive ESI mass spectrum of the L-tyrosine 1:1 H_2O, H_2SO_4/CH_3OH 1 x 10^{-3} M starting solution at pH=2. (b) Positive ESI mass spectrum of the rinsed precipitate obtained by microdroplets deposition.

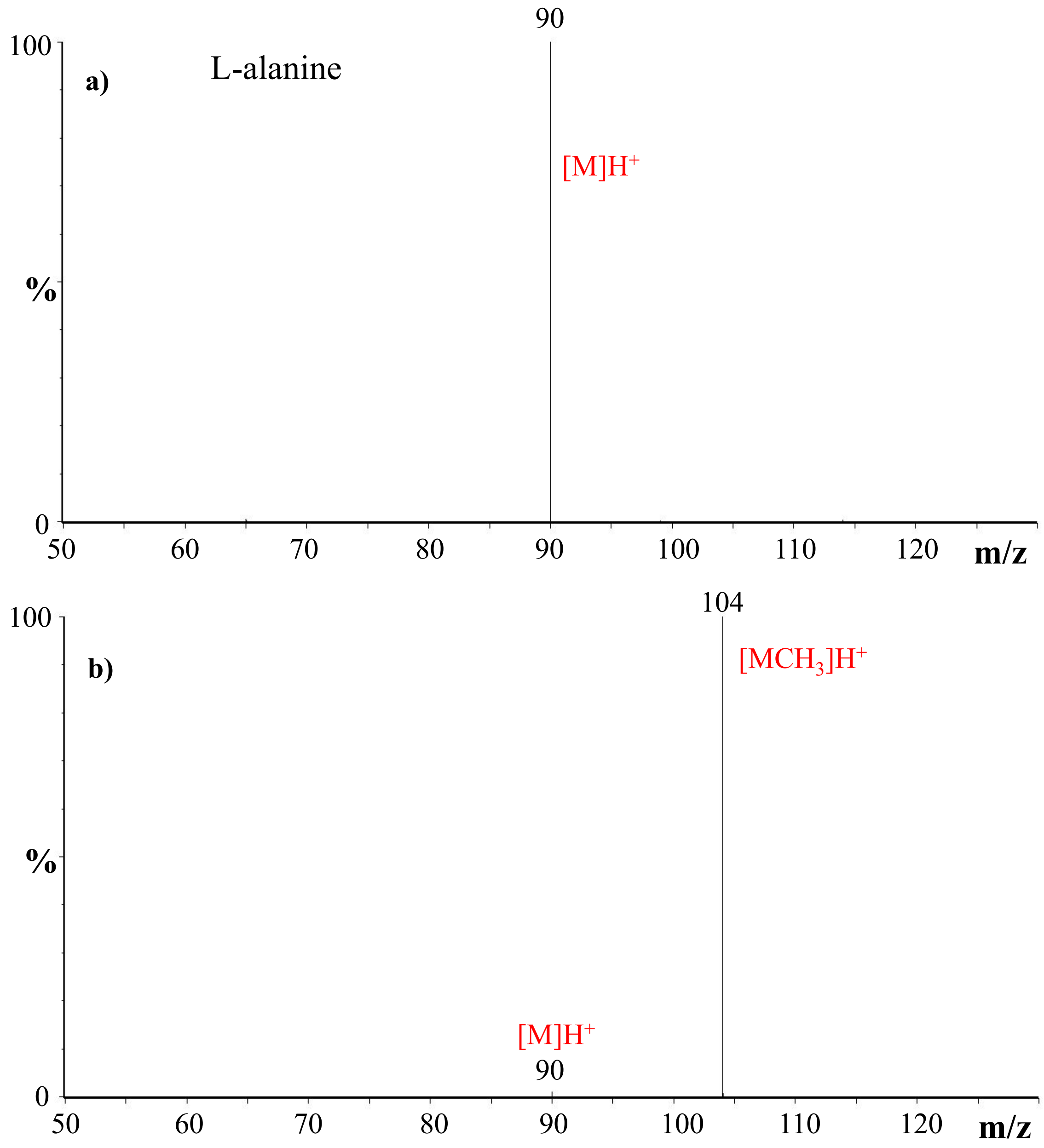

Figure S3: (a) Positive ESI mass spectrum of the L-alanine 1:1 H_2O, H_2SO_4/CH_3OH 1 x 10^{-3} M starting solution at pH=2. (b) Positive ESI mass spectrum of the rinsed precipitate obtained by microdroplets deposition.

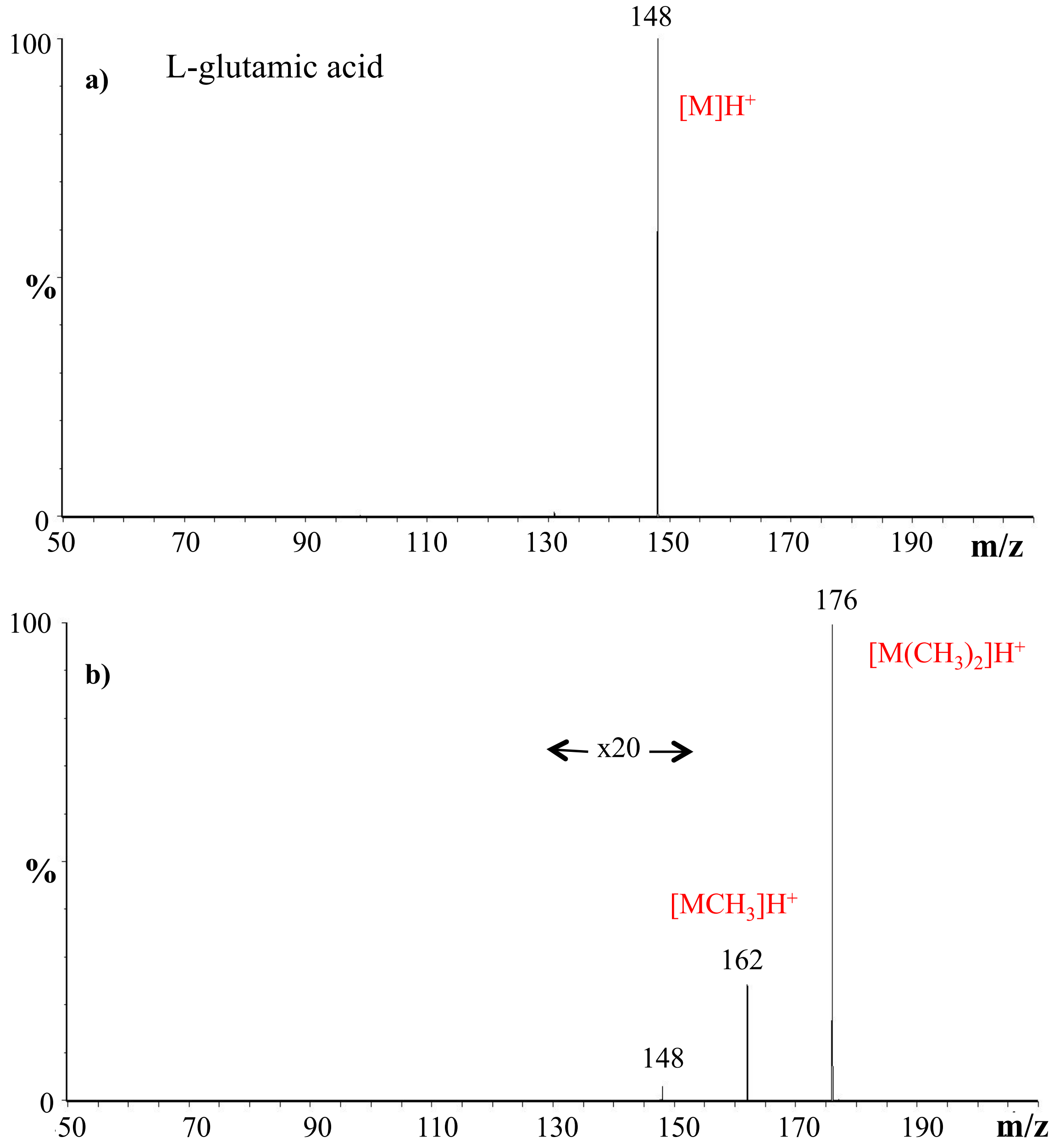

Figure S4: (a) Positive ESI mass spectrum of the L-glutamic acid 1:1 H_2O, H_2SO_4/CH_3OH 1 x 10^{-3} M starting solution at pH=2. (b) Positive ESI mass spectrum of the rinsed precipitate obtained by microdroplets deposition.

CID MASS SPECTRA

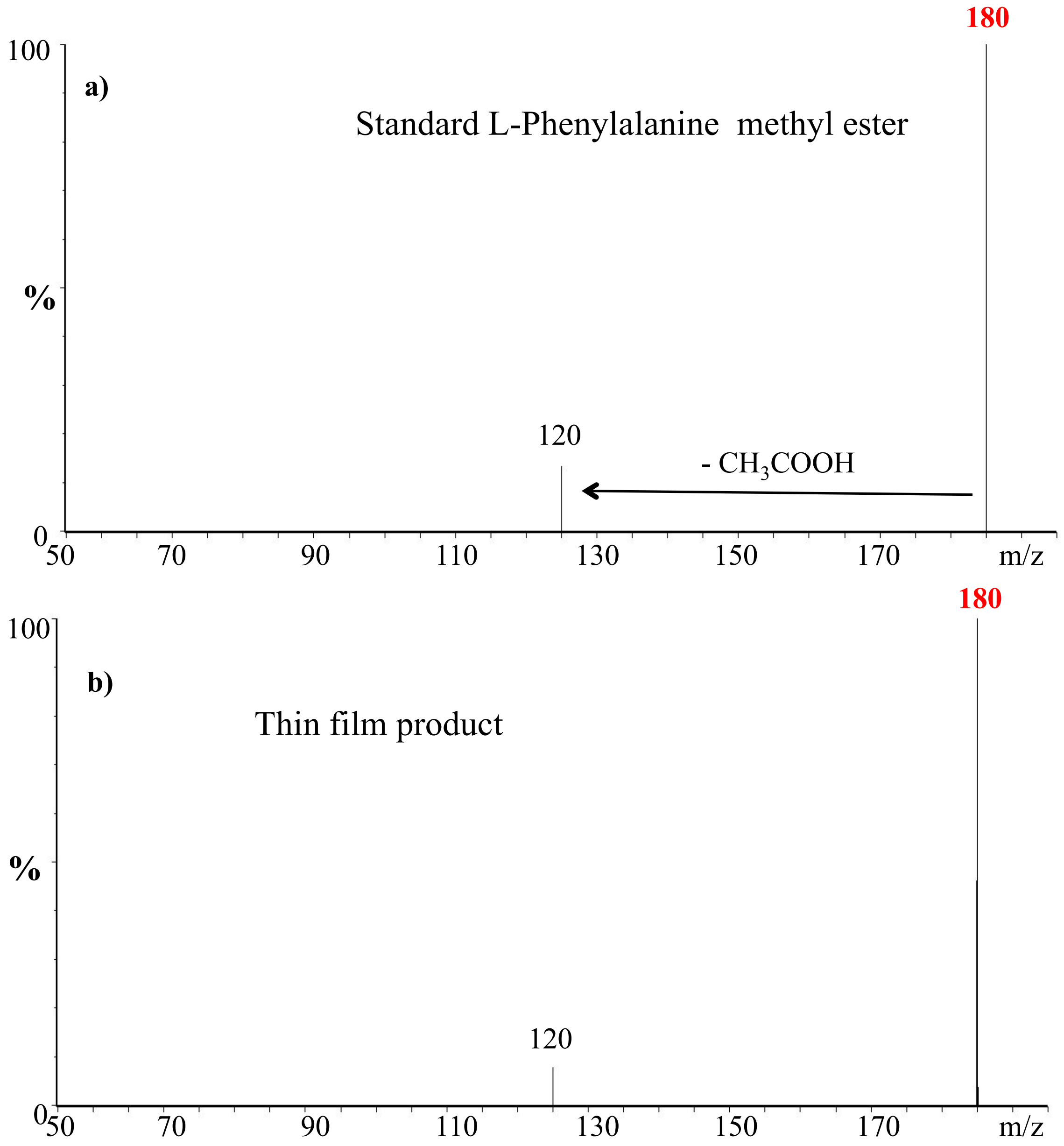

Figure S5: a) CID mass spectrum of standard L-phenylalanine methyl ester. b) CID mass spectrum of the thin film reaction product.

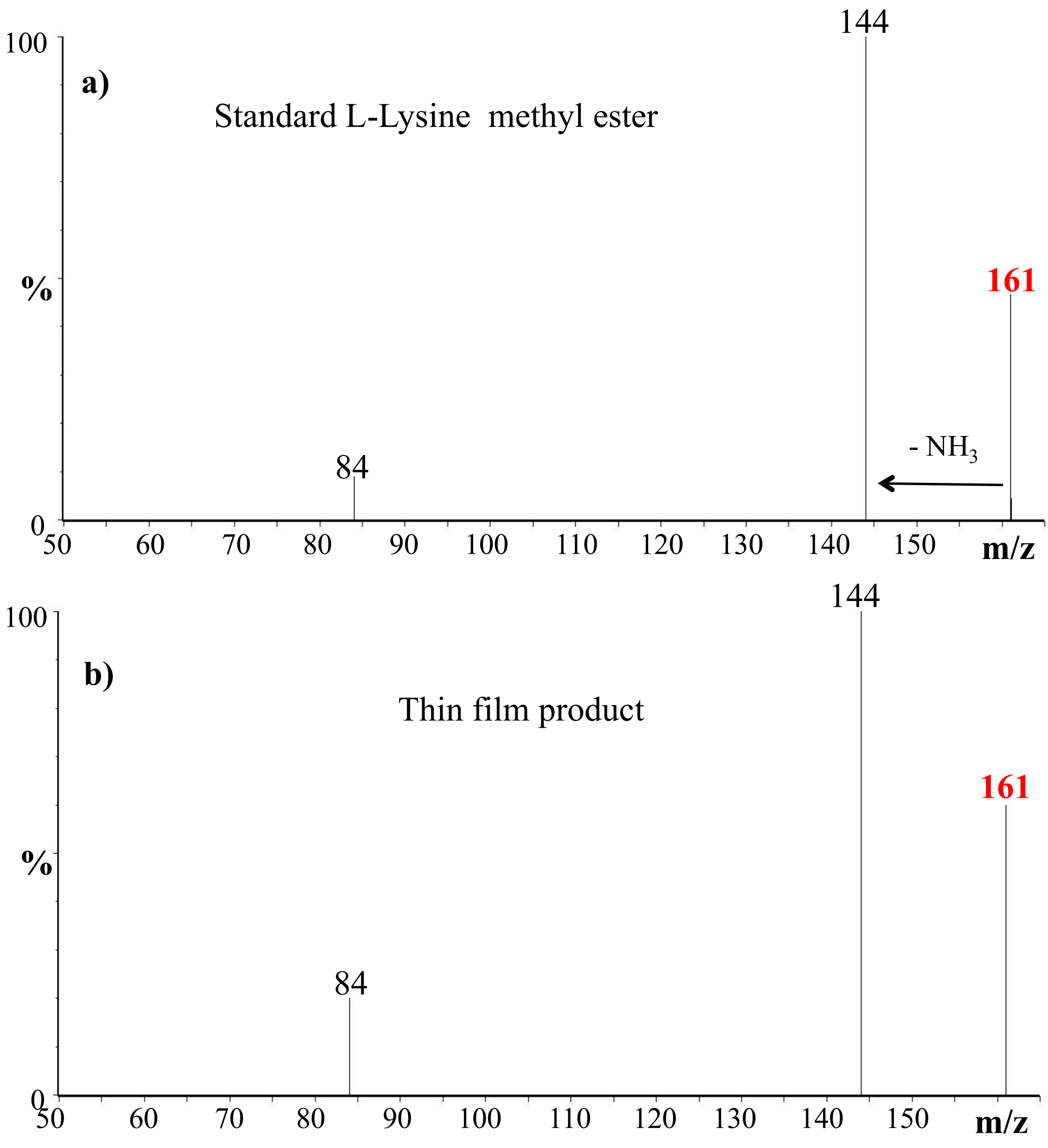

Figure S6: a) CID mass spectrum of standard L-lysine methyl ester. b) CID mass spectrum of the thin film reaction product.

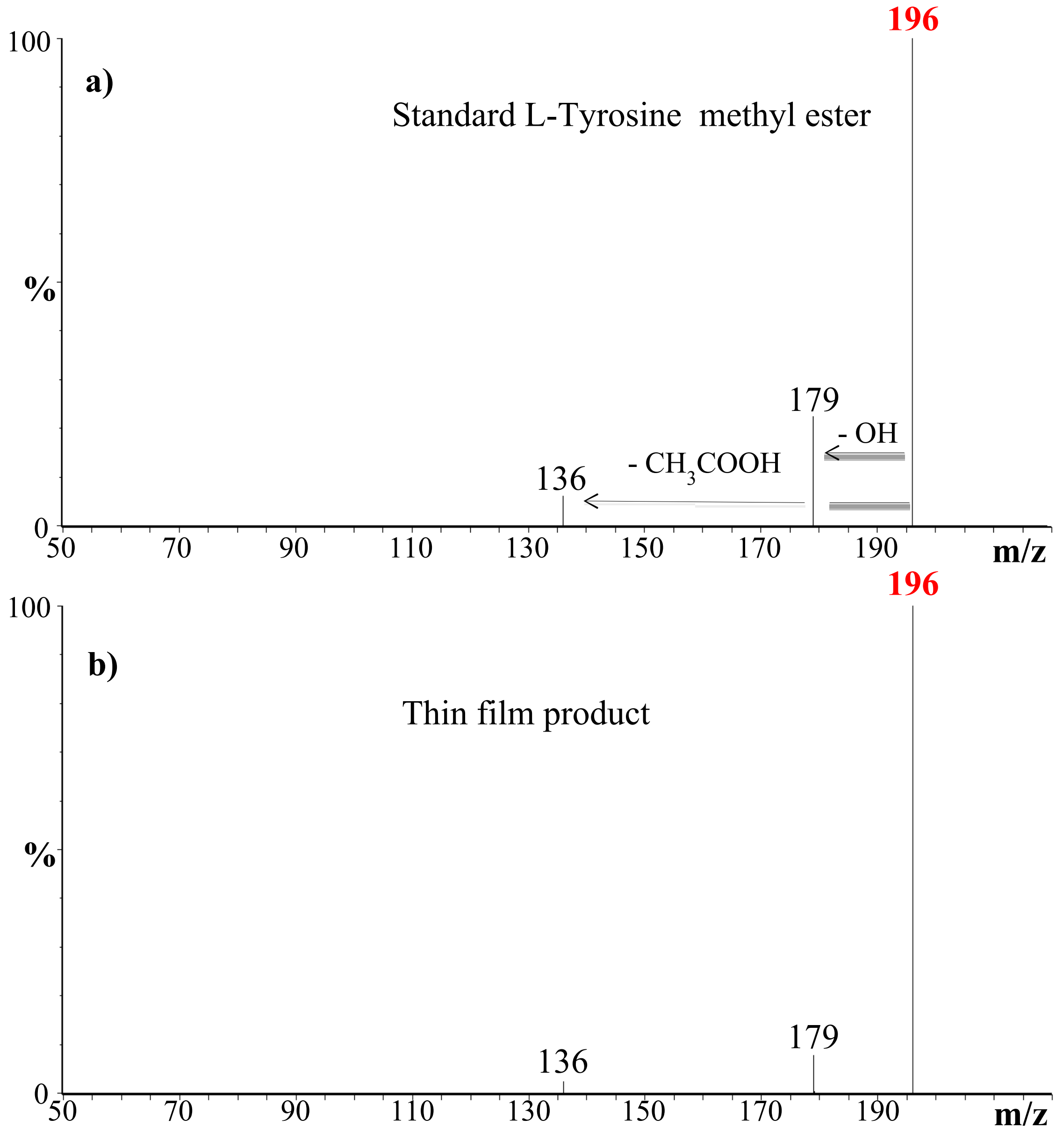

Figure S7: a) CID mass spectrum of standard L-tyrosine methyl ester. b) CID mass spectrum of the thin film reaction product.

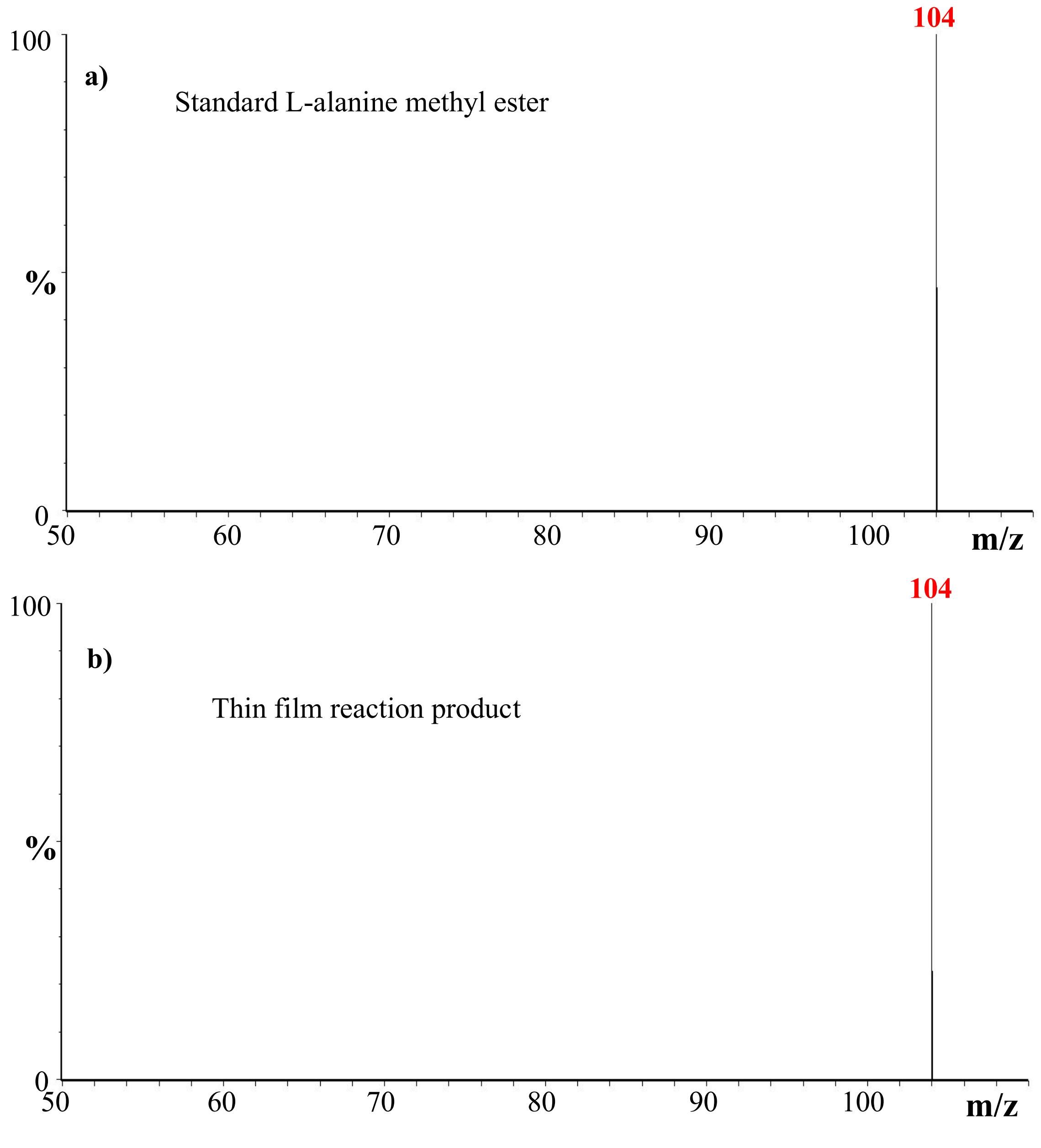

Figure S8: a) CID mass spectrum of standard L-alanine methyl ester. b) CID mass spectrum of the thin film reaction product.

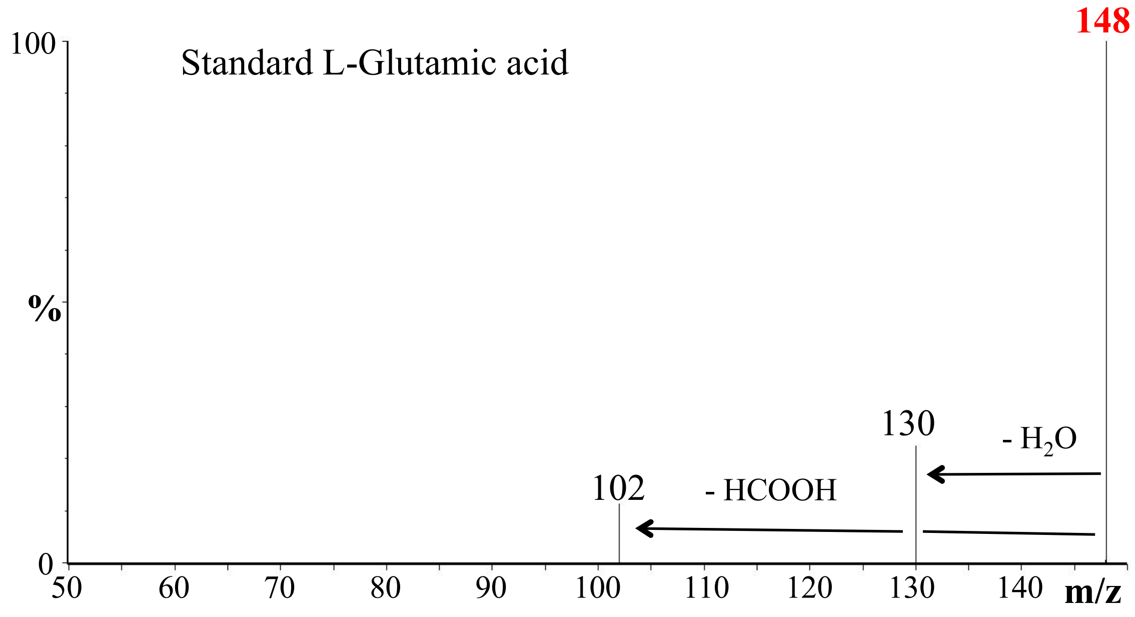

Figure S9: CID mass spectrum of standard L-glutamic acid.

IONIZATION CALIBRATION CURVES

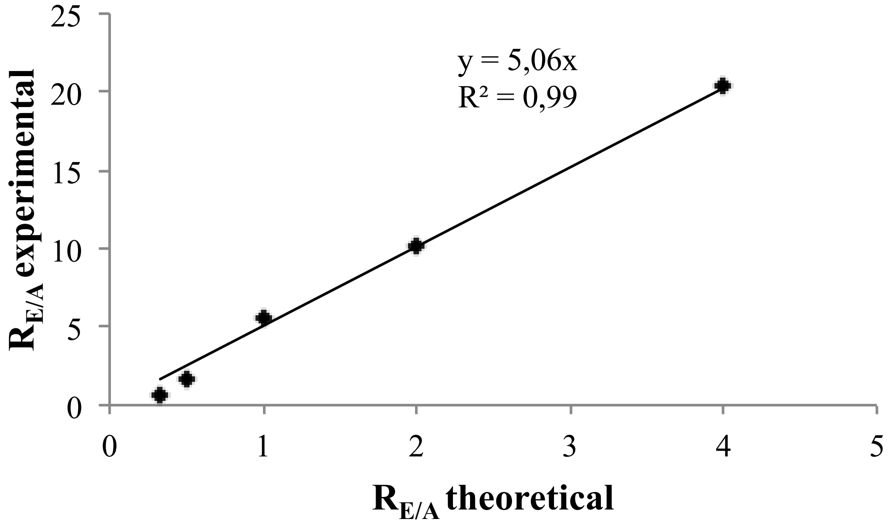

Figure S10. L-alanine methyl ester/amino acid ionization calibration curve.

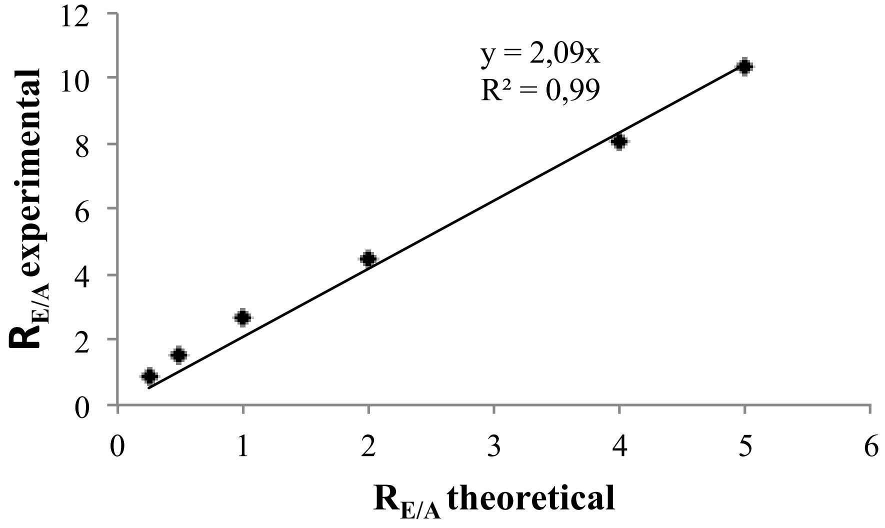

Figure S11: L-tyrosine methyl ester/amino acid ionization calibration curve.

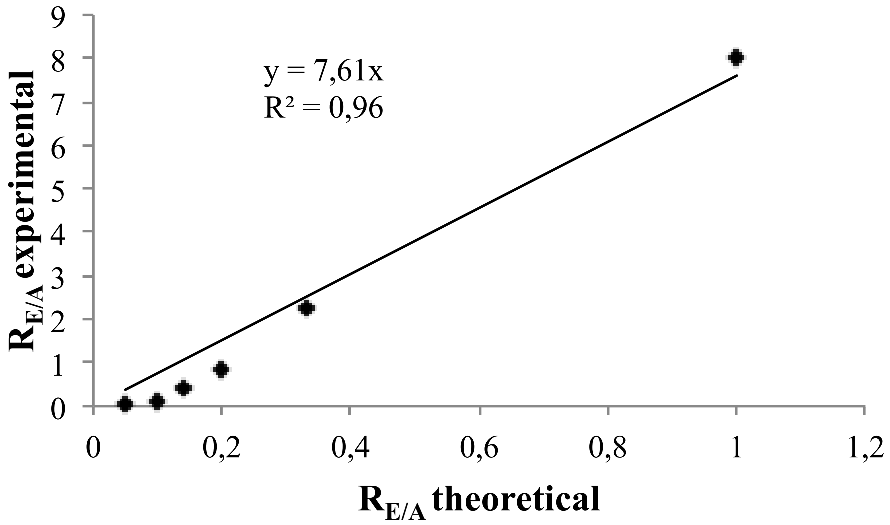

Figure S12: L-glutamic acid mono-methyl ester/amino acid ionization calibration curve.

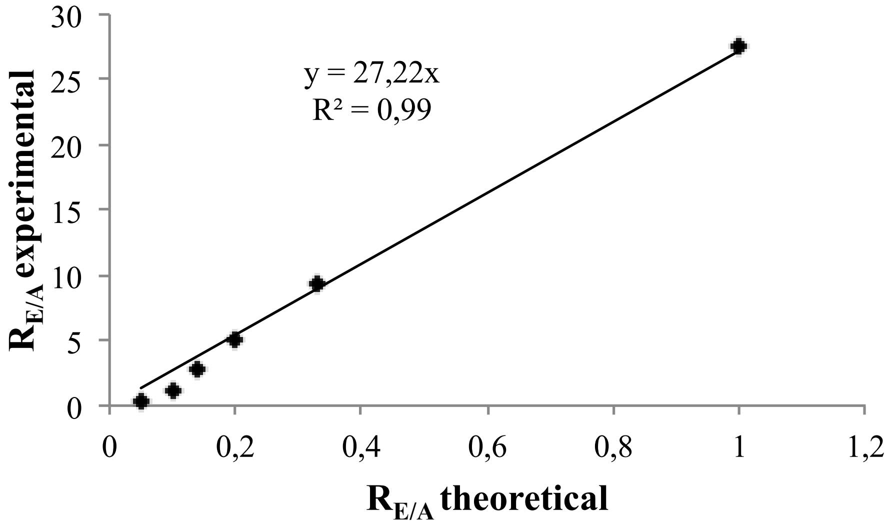

Figure S13: L-glutamic acid dimethyl ester/amino acid ionization calibration curve.
